# Post-Cessation Weight Gain Across Smoking Cessation Therapies: A Review of Secondary Analyses from the ZESCA, EVITA, and E3 Trials

**DOI:** 10.3390/ijerph22121819

**Published:** 2025-12-04

**Authors:** Audrey Lelièvre, Tetiana Zolotarova, Mark J. Eisenberg

**Affiliations:** 1 Centre for Clinical Epidemiology, Lady Davis Institute for Medical Research, Jewish General Hospital, Montreal, QC H3T 1E2, Canada; audrey.lelievre@ladydavis.ca (A.L.); tetiana.zolotarova@ladydavis.ca (T.Z.); 2 Faculty of Medicine, McGill University, Montreal, QC H3G 2M1, Canada; 3 Department of Epidemiology, Biostatistics and Occupational Health, McGill University, Montreal, QC H3A 1Y7, Canada; 4 Departments of Medicine and of Epidemiology, Biostatistics and Occupational Health, McGill University, Montreal, QC H3A 1Y7, Canada; 5 Division of Cardiology, Jewish General Hospital/McGill University, Montreal, QC H3T 1E2, Canada

**Keywords:** smoking cessation, bupropion, e-cigarette, varenicline, post-cessation weight gain

## Abstract

**Background:** Post-cessation weight gain is a barrier to smoking abstinence, yet evidence on the role of e-cigarettes in mitigating this remains limited. **Objective:** To examine weight-related effects of e-cigarettes in comparison with established cessation methods. **Methods:** We reviewed data from three cessation trials we conducted between 2005 and 2020. In ZESCA and EVITA, patients were randomized to bupropion or varenicline versus placebo. In the E3 trial, participants were randomized to counseling alone or with nicotine or non-nicotine e-cigarettes. Post hoc analyses assessed weight at 52 weeks for bupropion and varenicline, and 12 weeks for e-cigarettes. **Synthesis:** Abstinent individuals showed significant weight gain from baseline across the trials. In ZESCA and EVITA, abstinent participants gained more weight than intermittent and persistent smokers at 52 weeks (ZESCA: 4.8 vs. 2.0 vs. 3.0 kg, EVITA: 4.8 vs. 2.0 vs. −0.7 kg, respectively). Abstinent individuals gained more weight than persistent smokers (ZESCA: 3.4 kg, EVITA: 5.5 kg). In the E3 trial, abstinent participants with nicotine e-cigarettes gained more weight than those using non-nicotine e-cigarettes or counseling at 12 weeks (2.7 vs. 2.3 vs. 2.1 kg, respectively). **Conclusions:** Abstinent individuals experienced significant weight gain regardless of cessation treatment. Long-term effects of e-cigarettes on weight remain unclear.

## 1. Introduction

Although smoking cessation remains the most effective approach to reducing smoking-related morbidity, concerns about post-cessation weight gain are a common deterrent to quitting [1]. For many years, studies have identified quitting smoking as a key contributing factor to weight gain [2,3,4,5]. Over 80–90% of individuals who discontinue smoking experience post-cessation weight gain, with an average increase of 4.7 kg within the first year [5,6]. A study of telephone quit lines across the U.S. found that more than a quarter of calls involved concerns about post-cessation weight gain [7]. Although post-cessation weight gain may raise concerns, evidence indicates it does not substantially increase cardiovascular risk or reduce the health benefits of quitting smoking [8,9,10]. While some studies attribute weight fluctuations to the effects of nicotine withdrawal and changes in the energy intake-to-expenditure balance, the role of smoking cessation therapies has yet to be examined [11,12,13,14]. There are currently three pharmacotherapies approved for smoking cessation by the Food and Drug Administration, including nicotine replacement therapy, bupropion, and varenicline [15,16]. Although electronic cigarettes (e-cigarettes) use has not yet been approved for smoking cessation, they remain commonly utilized for quitting cigarette [17,18]. Our team conducted studies examining the efficacy of bupropion, varenicline, and e-cigarettes on smoking cessation [19,20,21]. These randomized clinical trials share similar protocols offering a valuable opportunity to compare the effects of e-cigarettes, bupropion, and varenicline on weight gain. This review summarizes the findings on post-cessation weight gain as observed in ZESCA, EVITA, and E3 trials [19,20,21,22].

## 2. Methods

We reviewed secondary analyses of weight change reporting 52-week outcomes of the use of bupropion (ZESCA) and varenicline (EVITA), and 12-week outcomes of e-cigarettes use (E3 trial). Full study details have been published previously, and the randomization procedure is outlined in Supplementary Materials S1–S3 [19,23,24,25,26,27]. In brief, participants included in the analyses received a 9 or 12-week course of treatment and completed all in-person follow-up visits where anthropometric measurements were obtained. Smoking status was self-reported and biochemically verified across all trials. Baseline characteristics and weight data were formerly presented in post hoc analyses as means, standard deviations (SD), medians, interquartile ranges (IQR), and percentages with corresponding 95% confidence interval. Multiple linear regression analyses were performed to assess the independent associations between baseline characteristics and 12-month weight outcomes in secondary analyses of the ZESCA and EVITA trials. In contrast, E3 trial weight analyses used a doubly robust causal inference framework, integrating propensity score estimation with outcome regression modeling to mitigate confounding. These analyses also included as-treated regression approaches using continuous exposure variables. Marginal structural models and generalized propensity score weighting were applied. Missing data were addressed through extreme gradient boosting imputation. Data from all three trials were integrated in this study.

## 3. Synthesis

Across all three trials, included participants were predominantly males with a mean age ranging from 51.7 to 55.0 years. At baseline the mean weight was 78.3 kg in ZESCA, 84.1 kg in EVITA, and 81.7 kg in the E3 trial. Study participants had been smoking for over 30 years on average, with more than 20 cigarettes smoked per day. More than 30% lived with another person who smoked. Less than one in six had a history of diabetes mellitus, while over a third had hypertension. In ZESCA and EVITA, majority of participants (>50%) had a history of hyperlipidemia (Table 1).

The ZESCA (Zyban as an Effective Smoking Cessation Aid for Patients Following Acute Coronary Syndrome) trial evaluated the effectiveness of bupropion compared with placebo among patients with elevated cardiovascular risks [19]. A total of 179 participants from the original cohort were included in a subsequent study assessing weight change among individuals attempting to quit smoking [25]. Participants were categorized as abstainers (*n* = 92), persistent smokers (*n* = 38), and intermittent smokers (*n* = 49) according to reported smoking status at follow-up. Individuals who reported abstinence at the 12-month follow-up were categorized as abstainers. Smoking abstinence was higher in the bupropion arm but not significantly different to placebo arm at 52 weeks (37.2% vs. 32.0%, *p* = 0.33) [19]. There was a decrease in mean daily cigarette consumption from baseline among both treatment arms (23.2 to 8.3 vs. 22.8 to 8.6) [19]. Findings indicated a median weight increase of 4.0 kg across all groups at 52 weeks. Individuals who were abstinent experienced more weight gain than intermittent and persistent smokers (4.8 kg vs. 2.0 kg vs. 3.0 kg, respectively) (Table 2). More than 40% of abstinent participants gained over 5 kg while 3.4% lost over 5 kg after one year [25]. Abstainers showed a significantly greater weight gain compared to persistent smokers at 52 weeks, with a mean difference of 3.4 kg (95% CI 1.4 to 5.4) [25].

EVITA (Varenicline for Smoking Cessation in Hospitalized Patients with Acute Coronary Syndrome), a second placebo-controlled trial, examined the effect of varenicline in a cohort of 302 participants across Canada and the United States [20]. Among those 302 participants, 172 met the criteria for inclusion in the analysis of weight outcomes. Participants were categorized into abstainers (*n* = 70), persistent smokers (*n* = 34), and intermittent smokers (*n* = 68). Similarly to ZESCA, individuals who reported abstinence at the 12-month follow-up were categorized as abstainers. Seven-day point prevalence abstinence was significantly higher in the varenicline group compared to placebo at 52 weeks (39.9% vs. 29.9%, 95% CI 0.01% to 21.44%) [22]. There was a reduction of over 50% daily cigarette consumption among both treatment arms (57.8% vs. 49.7%, 95% CI −3.1% to 19.4%) [22]. Findings indicated an overall mean weight gain of 2.6 kg at 52 weeks [26]. Individuals who were abstinent from smoking experienced more weight gain than intermittent smokers (4.8 ± 8.6 kg vs. 2.0 ± 8.9 kg). In contrast, persistent smokers lost on average 0.7 ± 7.4 kg (Table 2). Abstainers showed a significantly greater weight gain compared to persistent smokers at 52 weeks, with a mean difference of 5.5 kg (95% CI 2.3 to 8.8) [26]. Over 60% of abstinent participants gained more than 5% of their baseline weight [26].

The E3 trial (Evaluating the Efficacy of E-cigarettes Use for Smoking Cessation in the General Population) examined the effect of e-cigarettes on smoking cessation [21]. Overall, 376 participants were randomized to one of three treatment arms including the following: nicotine e-cigarettes (15 mg/mL) and counseling, non-nicotine e-cigarettes and counseling, or counseling alone [21]. Secondary weight analyses were conducted on 257 out of 376 individuals from the E3 trial [27]. Participants were categorized as quitting (*n* = 53) or returning to smoking (*n* = 204) at 12 weeks. Seven-day point prevalence abstinence was significantly higher for participants with nicotine e-cigarette plus counseling compared to counseling alone at 12 weeks (21.9% vs. 9.1%, 95% CI 4.0 to 21.6). Similarly, there was a greater reduction in daily cigarette consumption in the nicotine and non-nicotine e-cigarette plus counseling groups compared to the counseling alone group at 12 weeks (−12.6 vs. −10.6 vs. −7.0, respectively) [21]. Results indicate that participants who quit smoking experienced a significant increase in weight from baseline compared to those who returned to smoking (Table 3). Individuals who quit smoking using nicotine e-cigarette gained more weight than those randomized to non-nicotine e-cigarette (2.7 kg, 95% CI 1.5 to 3.8 vs. 2.3 kg, 95% CI 1.2 to 3.4) and counseling alone group (2.7 kg, 95% CI 1.5 to 3.8 vs. 2.1 kg 95% CI 0.5 to 3.6). Participants receiving only counseling who returned to smoking experienced a mean weight loss of 0.2 kg over the same period of time (Table 3).

## 4. Discussion

This comparison of the ZESCA, EVITA, and E3 trials suggests that individuals who achieve smoking cessation gain significantly more weight than those who continue to smoke. Results indicate an average of 5 kg are gained within the first year of quitting with bupropion and varenicline as cessation aids. E-cigarette use was associated with an average weight gain of 3 kg in the early months of abstinence, potentially indicating a relatively beneficial effect for weight mitigation compared to standard pharmacotherapy. Nicotine e-cigarettes were linked to greater post-cessation weight gain in comparison to counseling alone.

### 4.1. Smoking Behaviors, Nicotine Withdrawal, and Weight Gain

A 2025 observational study examined smoking initiation, cessation, and prevalence in the Japanese population, using national health data from 1986 to 2019, through an age–period–cohort framework [28]. Findings showed higher cessation probabilities among males and an overall increase in cessation with age [28]. Within an aging population, this suggests that the proportion of individuals experiencing post-cessation weight gain may increase. These results also underscore the importance of considering gender differences in smoking behaviors as they may influence post-cessation weight outcomes. Moreover, both the duration of smoking and the level of nicotine dependence may influence post-cessation weight gain [29,30]. Males historically began smoking earlier than females which may lead to stronger nicotine dependence and greater weight gain after quitting [28,31]. Evidence in the literature suggests that nicotine plays a primary role in the effect of smoking on weight [6,13,14]. It modulates body weight by acting on the hypothalamus, a central neuroendocrine structure involved in appetite regulation and energy homeostasis [14]. Nicotine stimulates the release of neurotransmitters and hormones (e.g., dopamine, serotonin, pro-opiomelanocortin…) which suppress appetite and increases resting metabolic rate, thereby reducing caloric intake [14,32]. Through its action on cholinergic and catecholaminergic receptors in the brain, nicotine influences lipid metabolism by increasing thermogenesis in adipose tissue, resulting in lipolysis [14,33]. Although the mechanisms underlying post-cessation weight gain remain unclear, the discontinuation of nicotine may lead to a reversal of its metabolic effects, contributing to weight increase [14].

#### 4.1.1. E-Cigarettes as Behavioral Substitutes for Cigarettes

Smoking is a deeply ingrained habit, often established over many years, which can make cessation particularly challenging. The use of e-cigarettes may help mitigate behavioral changes by preserving hand-to-mouth action and allowing individuals to maintain habitual breaks throughout the day. As adjustable devices, e-cigarettes may serve as effective transitional tools by allowing users to control nicotine levels and gradually taper intake [34,35]. This facilitates the reduction in cravings and withdrawal symptoms typically experienced with abrupt cessation or standard treatments [36,37]. As users progressively discontinue both smoking and vaping, they may be less inclined to replace hand-to-mouth behavior with increased food consumption. By targeting the behavioral cues of smoking, e-cigarettes could help minimize compensatory eating and the associated weight gain.

Although our data reflect short-term outcomes related to e-cigarette-associated weight gain, the observed increase is nearly half that reported with other cessation treatments. Given that the first three months following cessation are associated with the greatest weight gain, it remains possible that e-cigarettes may ultimately lead to less weight gain than other cessation methods [5].

#### 4.1.2. Weight Management Interventions

Novel intervention models have begun to integrate weight management into the cessation process. A randomized trial compared structured weight stability or weight loss interventions to low-intensity self-guided bibliotherapy in varenicline-assisted cessation [38]. Participants took part in the weight management program and behavioral intervention before beginning a 6-month course of varenicline [38]. Results showed that abstinent participants in both structured programs lost weight, whereas those in the self-guided group gained weight. A study tested a behavioral counseling approach that combined rewarding activities and skill-building with nicotine patches [39]. Among participants who remained abstinent at 26 weeks, there was no significant difference in weight gain between the intervention and standard counseling groups. While behavioral interventions show promise in managing post-cessation weight gain, new avenues of research explore pharmacological weight-management agents.

### 4.2. Future Directions

Emerging studies are investigating the use of Glucagon-like peptide-1 Receptor Agonists (GLP-1 RA) as adjunct therapies to support smoking cessation. GLP-1 is an incretin hormone produced by the L-cells of the small intestine in response to nutrient ingestion [40]. It plays a key role in the regulation of postprandial glucose levels and appetite by enhancing insulin secretion, inhibiting glucagon release, delaying gastric emptying, and promoting satiety [41]. Originally developed for the treatment of type 2 diabetes mellitus, GLP-1 receptor agonists (GLP-1RAs) have demonstrated substantial efficacy in reducing body weight among individuals without diabetes [42,43].

GLP-1RAs are being increasingly considered for their potential to support smoking cessation and manage associated weight gain, with early research also investigating their utility in the treatment of substance use disorders [44,45,46]. A pilot trial examined a six-week course of exenatide combined with nicotine replacement therapy for smoking cessation and weight gain prevention in prediabetic and overweight smokers [47]. While participants randomized to the exenatide arm experienced weight gain, it was 2.5 kg less than the weight gain observed in the placebo group. Exenatide has also been linked to reduced cravings and withdrawal symptoms in abstinent individuals [47]. It should be noted that exenatide has not yet been approved for weight loss and GLP-1RA treatments are typically evaluated over 16 to 104 weeks to assess efficacy [43]. Moreover, results from preclinical studies suggest that activation of GLP-1 receptors may enhance satiety and reduce aversive effects of nicotine by engaging the medial habenula–interpeduncular nucleus (MHb-IPN) neuronal pathway [48].

With ongoing research on the use of GLP-1 RAs for mitigating post-cessation weight gain, it may be important to identify patients who would benefit most from such interventions using individual level data. Substantial weight gain is relative to baseline weight, suggesting that GLP-1 RAs may be most appropriate for individuals at elevated risk of obesity. Targeting treatment to high-risk populations could help minimize long-term dependence on GLP-1 RA therapy.

### 4.3. Study Limitations

Firstly, an important limitation of the present study is that weight changes were reported in absolute terms, which may inadequately reflect the clinical relevance of weight gain. This approach might mask population-level patterns and heterogeneity across subgroups. Secondly, weight change was analyzed over differing follow-up durations, with varenicline and bupropion assessed over 52 weeks, and e-cigarettes over 12 weeks. This discrepancy hinders direct comparisons between interventions. Thirdly, it is important to note that the evidence presented in this review is derived from three post hoc analyses, which may introduce potential biases. Post hoc manipulation of data can be susceptible to confirmation bias, potentially leading to overestimated effects. Rigorous statistical measures were applied, as each subsequent analysis conducted appropriate statistical tests to minimize misestimation of effects. A fourth limitation is the reliance on self-reported measures. While common in smoking cessation studies due to the impracticality of continuous supervision, self-report may introduce systematic error through recall bias or social desirability. To mitigate this bias, however, we have routinely verified smoking status using a biochemical assessment of exhaled carbon monoxide.

## 5. Conclusions

Our review of the ZESCA, EVITA, and E3 trials weight analyses suggests that individuals who successfully quit smoking tend to experience greater weight gain compared to those who continue smoking. There is an average increase of 4–5 kg after 52 weeks of treatment with bupropion and varenicline. In comparison with standard pharmacotherapies, short-term effects of e-cigarette point to minimal effect on mitigation among abstinent individuals. Post-cessation weight gain should be considered in clinical guidance for smoking cessation. Incorporating strategies to mitigate weight gain at the start of a quit attempt may be beneficial. GLP-1 receptor agonists show promise in addressing post-cessation weight gain when used alongside conventional cessation strategies; however, further trials are warranted to establish their effectiveness. Pipeline research should consider combining smoking cessation with weight management interventions. Additional studies are required to understand long term effects of e-cigarette for smoking cessation on weight.

## Figures and Tables

**Table 1:** Baseline characteristics of study participants (ZESCA, EVITA, and E3 trial).

Characteristics	ZESCA (*n* = 179)	EVITA (*n* = 172)	E3 Trial (*n* = 257)
	Demographic characteristics		
Age, mean (SD)	53.9 (10.0)	57.1 (9.3)	51.7 (12.0)
Male, %	84.0	77.2	52.5
	Anthropometric characteristics		
Weight, mean (SD)	78.3 (17.7)	84.1 (15.1)	81.7 (18.3)
	Smoking characteristics		
No years smoked, mean (SD)	33.7 (12.0)	20.8 (10.8)	35.3 (13.7)
No cigarettes/day, mean (SD)	23.2 (10.4) *	28.5 (8.0) *	21.3 (11.7) ^†^
Other smokers at home, %	32.6	42.8	29.9
	Clinical characteristics		
Hyperlipidemia	50.0	62.7	37.0
Hypertension	36.5	44.2	31.5
Diabetes Mellitus	15.1	16.6	16.7

Abbreviations: SD—standard deviation. * In the past year. ^†^ In the past 10 years.

**Table 2:** Weight change from baseline to 52-week follow-up (ZESCA and EVITA trials).

Trial	Sample Size, *n*	Study Arms	Weight Change (kg), Mean (SD)/Median (IQR)		
			At 52 Weeks		
			Persistent Smokers	Intermittent Smokers	Abstainers
**ZESCA**	179	Bupropion—Placebo *	3.0 ^†^ (−0.8, 6.0) (*n* = 38)	2.0 ^†^ (−2.0, 5.0) (*n* = 49)	4.8 ^†^ (1.0, 8.6) (*n* = 92)
**EVITA**	172	Varenicline—Placebo *	−0.7 (7.4) (*n* = 34)	2.0 (8.9) (*n* = 68)	4.8 (8.6) (*n* = 70)

* Pooled data. ^†^ Median weight-change.

**Table 3:** Weight change from baseline to 12-week follow-up (E3 trial).

Trial	Sample Size, *n*	Study Arms	Weight Change (kg), Mean (95% CI)	
			At 12 Weeks	
			Smokers	Abstainers
**E3 trial**	257	Nicotine EC + counseling	0.3 (−0.4, 1.1) (*n* = 76)	2.7 (1.6, 3.8) (*n* = 25)
		Non-nicotine EC + counseling	0.1 (−0.6, 0.8) (*n* = 72)	2.3 (1.2, 3.4) (*n* = 19)
		Counseling	−0.2 (−0.8, 0.3) (*n* = 56)	2.0 (0.5, 3.6) (*n* = 9)

## Data Availability

This review is based exclusively on published, publicly available data. No individual participant data were collected or analyzed. All data sources utilized in this study are appropriately cited within the manuscript.

## Supplementary Materials

The following supporting information can be downloaded at: https://www.mdpi.com/article/10.3390/ijerph22121819/s1, Supplementary Material S1: Randomization and Follow-up of Study Patients in ZESCA Trial; Supplementary Material S2: Randomization and Follow-up of Study Patients in EVITA Trial; Supplementary Material S3: Randomization and Follow-up of Study Patients in E3 Trial.
