# Supplementary material for: Post-Cessation Weight Gain Across Smoking Cessation Therapies: A Review of Secondary Analyses from the ZESCA, EVITA, and E3 Trials
Source: Int J Environ Res Public Health. 2025 Dec 4;22(12):1819. doi: 10.3390/ijerph22121819 (PMC12732817; doi:10.3390/ijerph22121819)

Supplementary Materials

Content **Page**

1. Supplementary Material S1: Randomization and Follow-up of Study Patients in ZESCA Trial 3
2. Supplementary Material S2: Randomization and Follow-up of Study Patients in EVITA Trial 4
3. Supplementary Material S3: Randomization and Follow-up of Study Patients in E3 Trial 5

Supplementary Material S1. Randomization and Follow-up of Study Patients in ZESCA Trial

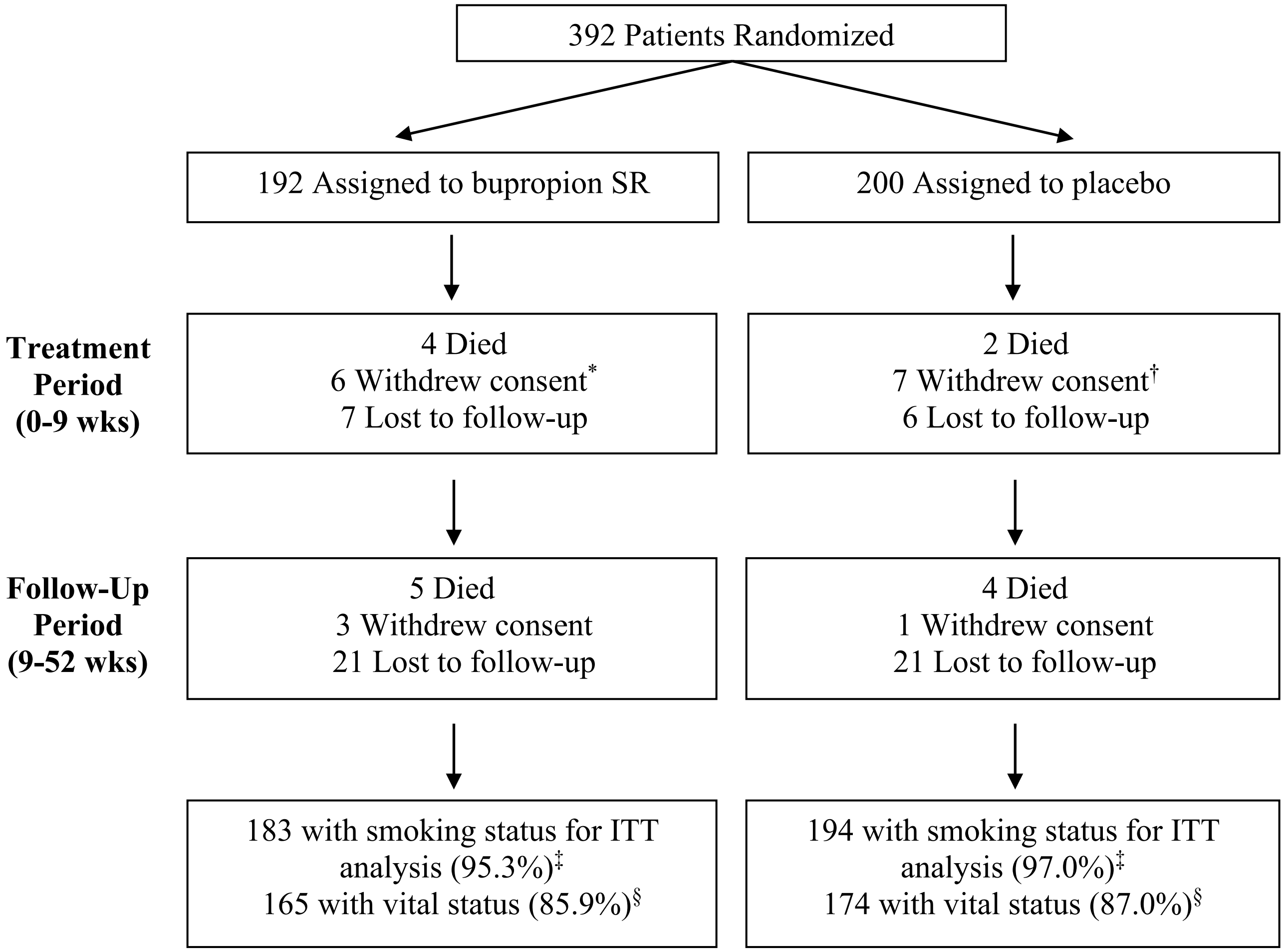

* Two patients who withdrew consent did not receive treatment.
† One patient who withdrew consent did not receive treatment.
‡ Includes all patients except those who died. For the ITT analysis, patients who were lost to follow-up or withdrew were assumed to have gone back to smoking at baseline rates.
§ Of the 392 patients enrolled, 3.8% died and 22.2% were lost to follow-up. Among those lost to follow-up, vital status was obtained via medical charts for 8.7% of patients. Vital status was obtained for 10 patients in the bupropion group and 9 patients in the placebo group among those who did not return for clinical follow-up.
Abbreviations: ITT, intention-to-treat.

Supplementary Material S2. Randomization and Follow-up of Study Patients in EVITA Trial

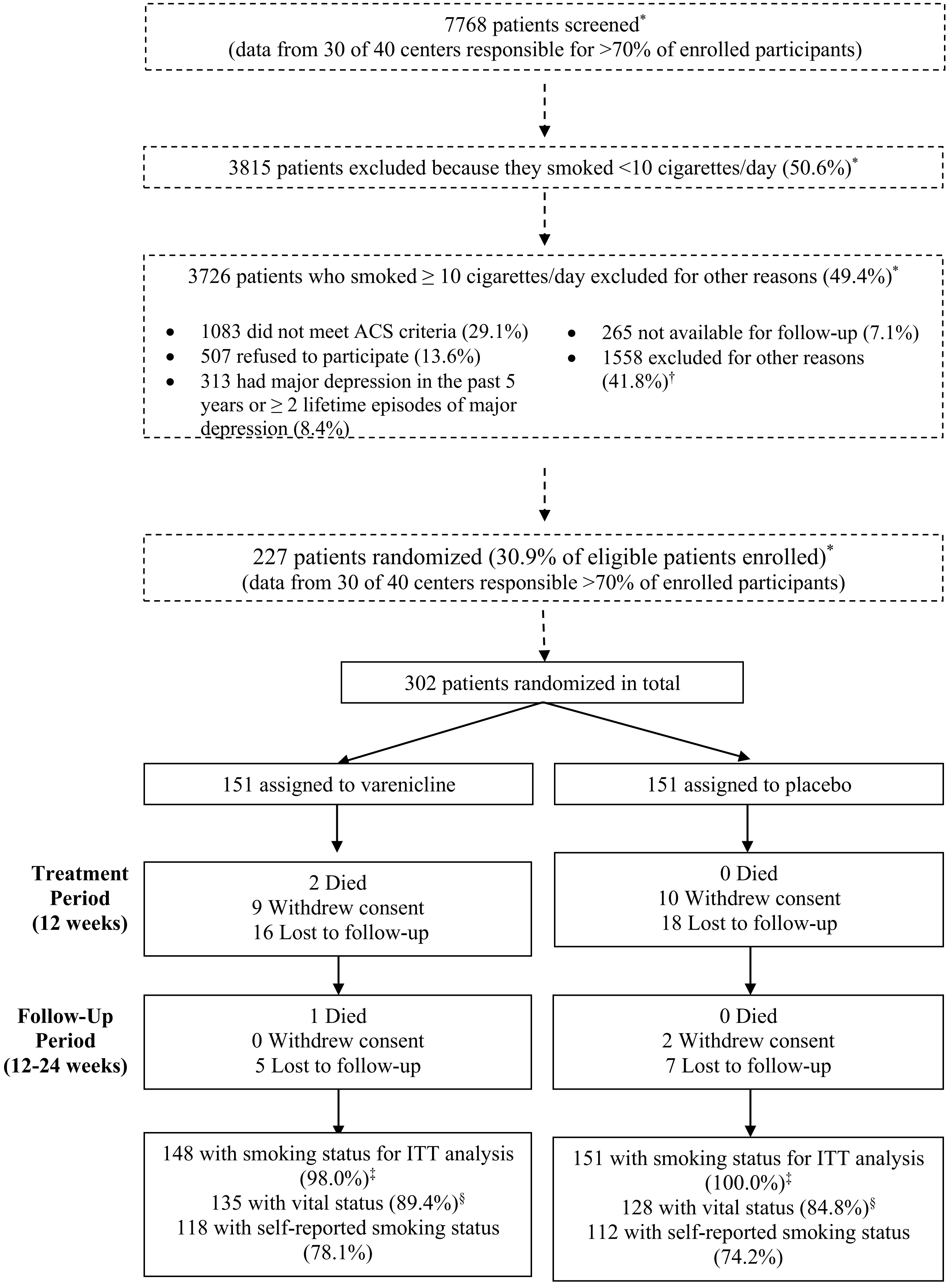

Supplementary Material S3. Randomization and Follow-up of Study Patients in E3 Trial

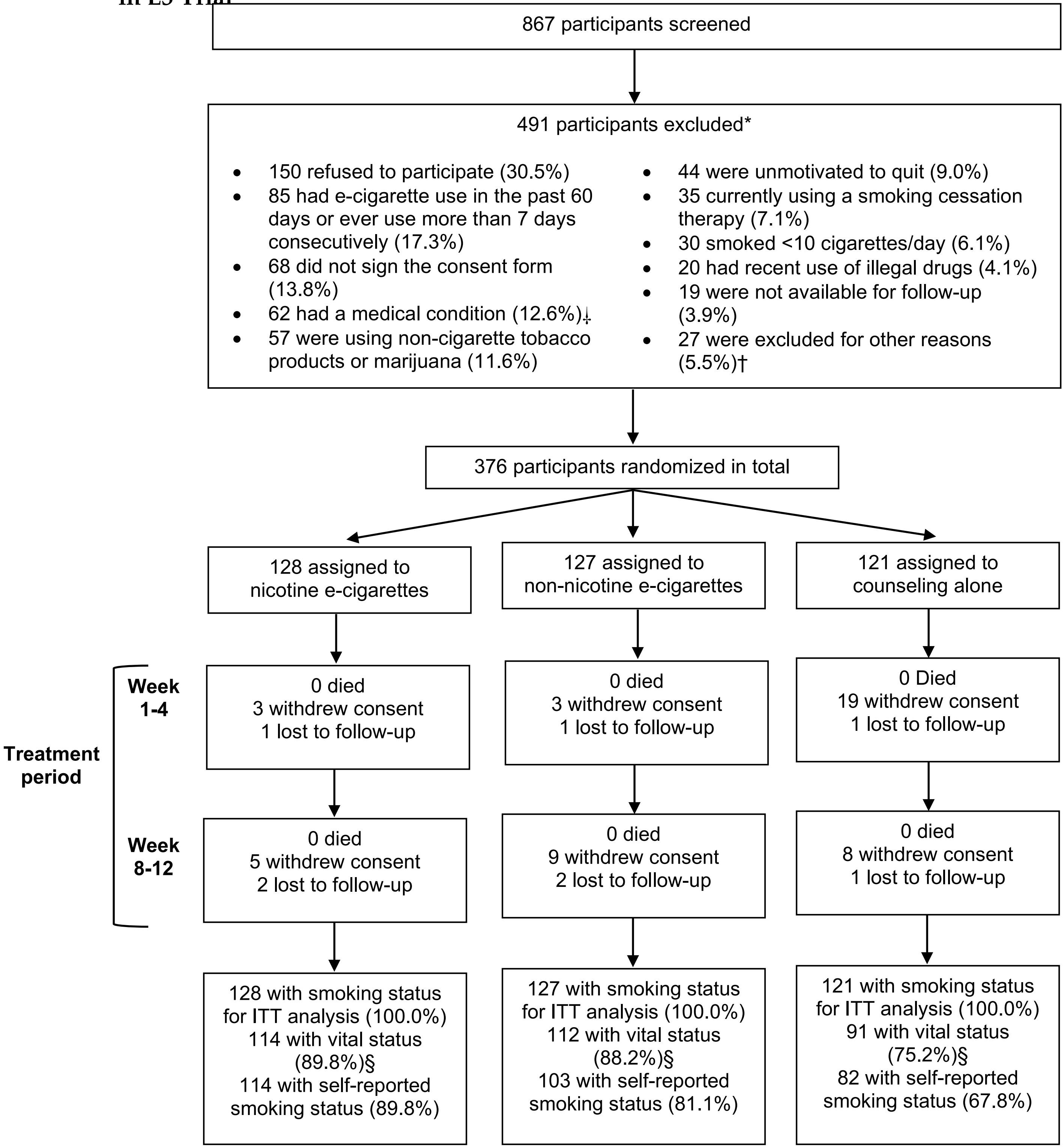

Supplement: Supplementary file 1 [file ijerph-22-01819-s001.zip › ijerph-3931681-supplementary.pdf]
